# Chronic Rhinotillexomania Leading to Multiple Infectious Sequelae in a 66-Year-Old Female

**DOI:** 10.7759/cureus.9856

**Published:** 2020-08-18

**Authors:** Kunal Shah, Praveen Sankrithi, Sanket Shah, Mark A Smith

**Affiliations:** 1 College of Osteopathic Medicine, Kansas City University of Medicine and Biosciences, Kansas City, USA; 2 Hospice & Palliative Medicine, Catholic Health Initiatives St. Vincent Medical Center-North, Little Rock, USA

**Keywords:** rhinotillexomania, nose-picking

## Abstract

Rhinotillexomania, more commonly known as nose-picking, is a body-focused repetitive behavior that involves compulsive picking of the nares. While nose-picking in general is a common and largely unharmful practice worldwide, severe complications from chronic nose-picking do exist. This case highlights a 66-year-old female with a longstanding history of nose-picking and nose blowing who presented to the emergency department with acute delirium and various bodily complaints. She was subsequently admitted multiple times for recurrent sepsis, meningitis, endocarditis, cystitis, and discitis with each culture positive for methicillin-sensitive *Staphylococcus aureus* (MSSA) with identical antibiotic sensitivities. Further imaging failed to reveal an underlying greater pathology. Intranasal swab revealed identical culture and sensitivity results as previous blood, CSF, and urine cultures and was thus hypothesized to be the source of infection. This case aims to elucidate the harmful effects of nose-picking as well as the importance of prevention, diagnosis, and management.

## Introduction

Nose-picking is an almost universal practice in adults with 91% of adult respondents to the Rhinotillexomania Questionnaire acknowledging digitalization of the nares as an active and regular behavior [1-4]. In addition, the average person picks their nose four times a day [1]. Picking of the nose is often a required and accepted behavior and, if limited, is without serious sequelae, serving to partially empty the nasal passages of obstructing nasal secretions, inhaled debris, and potentially pathogenic organisms. However, some cases of chronic nose-picking can be attributed to a condition known as Empty Nose Syndrome, an iatrogenic condition which results in the sensation of nasal congestion with a clinical examination consistent with atrophic or surgically absent turbinates [5-9]. These patients typically suffer from chronic atrophic rhinitis further propagating the compulsion of nose-picking.

Rhinotillexomania can result in minor disruptions of midline structures with the potential for routine healing if the inciting picking stimulus is aborted. Extreme cases of continued digital trauma can cause clinically significant epistaxis from the exposure of the underlying Kiesselbach’s vascular plexus which serves as the terminus of the sphenopalatine artery branch of the maxillary artery from the external carotid artery, the greater palatine branch of the maxillary artery from the external carotid artery, the superior labial branch of the facial artery from the external carotid artery, and the anterior ethmoidal branch from the internal carotid artery [5].

There are few deleterious effects of nose-picking, most of which are confined to the nares. However, the more severe cases are exceedingly rare and largely unreported in the literature. Some cases of nasal septal perforations are due to the digital trauma from chronic nose-picking, causing erosion through the nasal septal cartilage [3]. The literature also suggests that nose-picking increases rates of bacterial respiratory infections with pneumococcal species which may lead to pneumonia, a major cause of death worldwide [6]. According to several studies, nose pickers are significantly more likely to carry *Staphylococcus aureus* in the nares [2]. Mild epistaxis and nasal vestibulitis and folliculitis secondary to* Staphylococcus aureus* are also commonly attributed to nose-picking. Furthermore, sinus and nasal skin infections can lead to septic cavernous sinus thrombosis, a rare and life-threatening condition caused by the formation of a blood clot in the cavernous sinus of the brain. With many possible avenues for distant spread of infection, we discuss a novel case of a 66-year-old female with a history of chronic nose-picking who presented to the emergency department with recurrent sepsis, meningitis, endocarditis, discitis, and cystitis refractory to IV antibiotics.

## Case presentation

The patient is a 66-year-old Caucasian female with a history of well-controlled non-insulin dependent Type 2 diabetes mellitus, right total knee arthroplasty in 2012, and left total knee arthroplasty in 2014 who was initially referred to otolaryngology for evaluation of chronic rhinorrhea. She reported a greater than 30-month history of daily and repetitive intranasal tissue manipulation coupled with alternating daily use of over-the-counter oxymetazoline and phenylephrine nasal spray preparations. The physical examination revealed dry nasal mucosa with a left inferior bone spur along with intranasal scarring bilaterally over the anterior nasal septum likely secondary to excessive tissue use. For rhinitis medicamentosa, she was treated with a tapering dose pack of prednisone, intranasal flunisolide spray, and the directive to discontinue all over-the-counter medicated nose sprays. The patient did not return for follow-up evaluation.

The patient required hospitalization 26 months later for evaluation of a three-week history of progressive posterior neck pain and headache associated with nausea, and asymptomatic palpitations without febrility, vomiting, or mental aberrations. Blood, urine, and cardiac workup revealed no significant abnormalities. Chest and brain imaging was inconclusive. Neurologic consultation confirmed cervicalgia secondary to osteoarthritis with the degenerative joint disease of the cervical spine with cervical paraspinous muscle spasm. The head and neck discomfort resolved with physical therapy and acetaminophen. Lumbar puncture was declined by the patient who was discharged within 48 hours of admission.

The patient required another hospitalization six weeks later for evaluation of an acute onset and rapidly progressing dyspepsia with nausea and vomiting, febrility, confusion, disorientation, slurred incoherent speech, and atrial fibrillation with rapid ventricular response requiring cardiology and neurology consultation and medical intensive care unit (MICU) admission.

Lumbar puncture on the second day of hospitalization revealed RBC count of 5,220/mm3, WBC of count 5850/mm3, segmented neutrophils 93%, lymphocytes 7%, no cells observed for cytology, glucose 169 mg/dl, and total protein 127 mg/dl.

**Figure 1:**
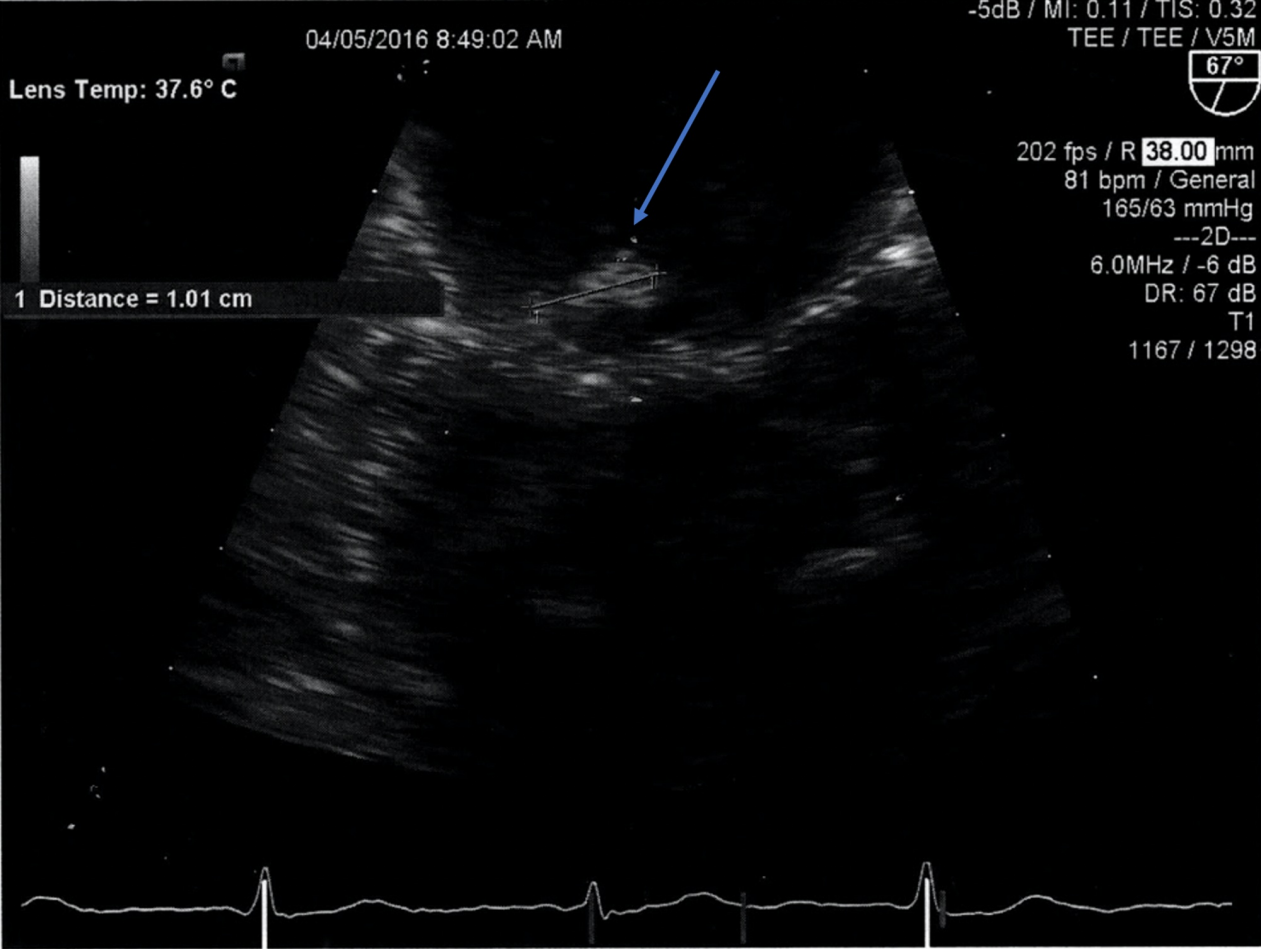
Transesophageal echocardiography Transesophageal echocardiography showing a moderate size (1.0 x 0.45 cm) mobile vegetation on the anterior leaflet (A3 scallop) of the mitral valve (blue arrow) with mild mitral valve regurgitation

Two blood cultures, urine cultures, and cerebrospinal fluid cultures revealed methicillin-sensitive *Staphylococcus aureus *(MSSA) with identical sensitivities. The patient developed right lateral elbow erythema, swelling, calor, and dolor with cellulitis and olecranon bursitis. The elbow and forearm had fullness noted in the region of the olecranon bursa with no underlying lytic or blastic bone lesions and no joint effusion. Ceftriaxone, vancomycin, and doxycycline were discontinued and nafcillin was initiated to better target MSSA. The specific sensitivities are detailed in Table 1. The patient manifested recurrent episodes of asystole of up to 10 seconds and required temporary pacemaker placement and discontinuation of amiodarone therapy. Transesophageal echocardiography revealed native mitral valve endocarditis with a 1 cm anterior leaflet vegetation with moderate mitral regurgitation, moderate aortic insufficiency, and patent foramen ovale (Figure 1). The right elbow pain and swelling clinically resolved within eight days with orthopedic surgery consultation resulting in no orthopedic intervention. The patient progressed to respiratory failure requiring Bilevel Positive Airway Pressure (BIPAP) ventilatory support with increasing obtundation. Referral to hospice was declined by the patient’s health care proxy. The temporary pacemaker was removed and the BIPAP ventilatory support was weaned and discontinued. The leukocytosis resolved and the patient was discharged to an acute rehabilitation hospital (ARH) for completion of intravenous nafcillin therapy.

**Table 1:** MSSA positive blood, cerebrospinal fluid, urine, and T4-5 disc sensitivities MSSA, methicillin-sensitive *Staphylococcus aureus*; R, resistant; S, sensitive

Drug	Sensitivity
Penicillin	R
Oxacillin	S
Gentamicin	S
Levofloxacin	S
Erythromycin	S
Clindamycin	S
Linezolid	S
Vancomycin	S
Tetracycline	S
Rifampin	S
Trimethoprim/Sulfa	S
Ciprofloxacin	S

The patient presented to the ED from the acute rehabilitation hospital four weeks later for symptomatic palpitations and hypokalemia without leukocytosis or febrility which promptly responded to treatment with intravenous and oral potassium replacement therapy and the initiation of beta-blockade therapy with no findings indicative of acute coronary syndrome. The patient was discharged to return to the ARH.

The patient required another hospitalization two weeks later after being admitted from the ARH for diarrhea, profound hypokalemia, generalized weakness, dehydration, and hypomagnesemia without leukocytosis or febrility with two serial stool sample specimens both negative for *C. difficile* toxin A and B. Gastroenterology consultation confirmed acute viral gastroenteritis with the patient promptly responding to intravenous fluid (IVF) hydration, oral and intravenous potassium and magnesium replacement therapy, probiotic therapy, and cholestyramine therapy. The patient was discharged home with home health where she manifested persistent anorexia, dyspepsia, and weight loss. She would later admit that she continued to excessively digitally manipulate her nares with a finger covered with tissue paper multiple times daily exhausting the supply of a medium-sized paper tissue box containing 65 tissues approximately every three days.

The patient required another hospitalization four weeks later for progressive intractable nausea, vomiting, increasing weight loss with a BMI of 17.8 kg/m2, and increasing generalized weakness with infrequent loose stools without febrility, chest pain, or palpitations. Her evaluation revealed dehydration and profound hypokalemia with a serum potassium of 1.9 millimoles/liter requiring IVF hydration and oral and intravenous potassium replacement for treatment. Two repeat blood cultures revealed MSSA with identical sensitivities to the blood cultures obtained 10 weeks earlier. The patient was discharged to a long term care facility for subacute care to receive six weeks of intravenous nafcillin therapy. 

During the subacute long term care facility admission for the administration of six weeks of intravenous nafcillin therapy, nasal swab bacterial culture of the right nares revealed MSSA with identical sensitivities to the two blood cultures, urine cultures, and cerebrospinal fluid cultures obtained 12 weeks earlier and the two blood cultures obtained two weeks earlier. Referral to otolaryngology revealed right anterior septum superficial excoriations and abrasions with no erythema or purulence. They initiated treatment with mupirocin ointment to the right anterior septum three times a day for seven days followed by the application of saline nasal gel to the anterior septum with the specific directive to discontinue all applications of paper tissue into the nares.

The patient required another hospitalization eight weeks later for evaluation of progressive weakness, right knee swelling and pain, left hip and proximal femur pain, and right neck pain and stiffness with chest x-ray (CXR) revealing no acute cardiopulmonary disease. Nuclear medicine leukocyte scan revealed no abnormal tracer accumulations in the head, chest, abdomen, or pelvis with no sites of acute infection or inflammation identified. Laboratory testing revealed a leukocytosis of 21.06 K/mm3 and at admission the patient was provided with empiric vancomycin and Levaquin therapy. The two blood and cerebrospinal fluid (CSF) cultures showed similar sensitivities as the previous hospitalizations. The patient was discharged home with home health to complete a 10-day course of oral Levaquin therapy

The patient required another hospitalization 10 days later for recurrent generalized weakness, anorexia, cachexia, and debility with dehydration. She also had relative hypotension with mild leukocytosis and two negative blood cultures. She promptly responded to treatment with IVF hydration. The patient was discharged home with home health on oral levaquin therapy. 

**Figure 2:**
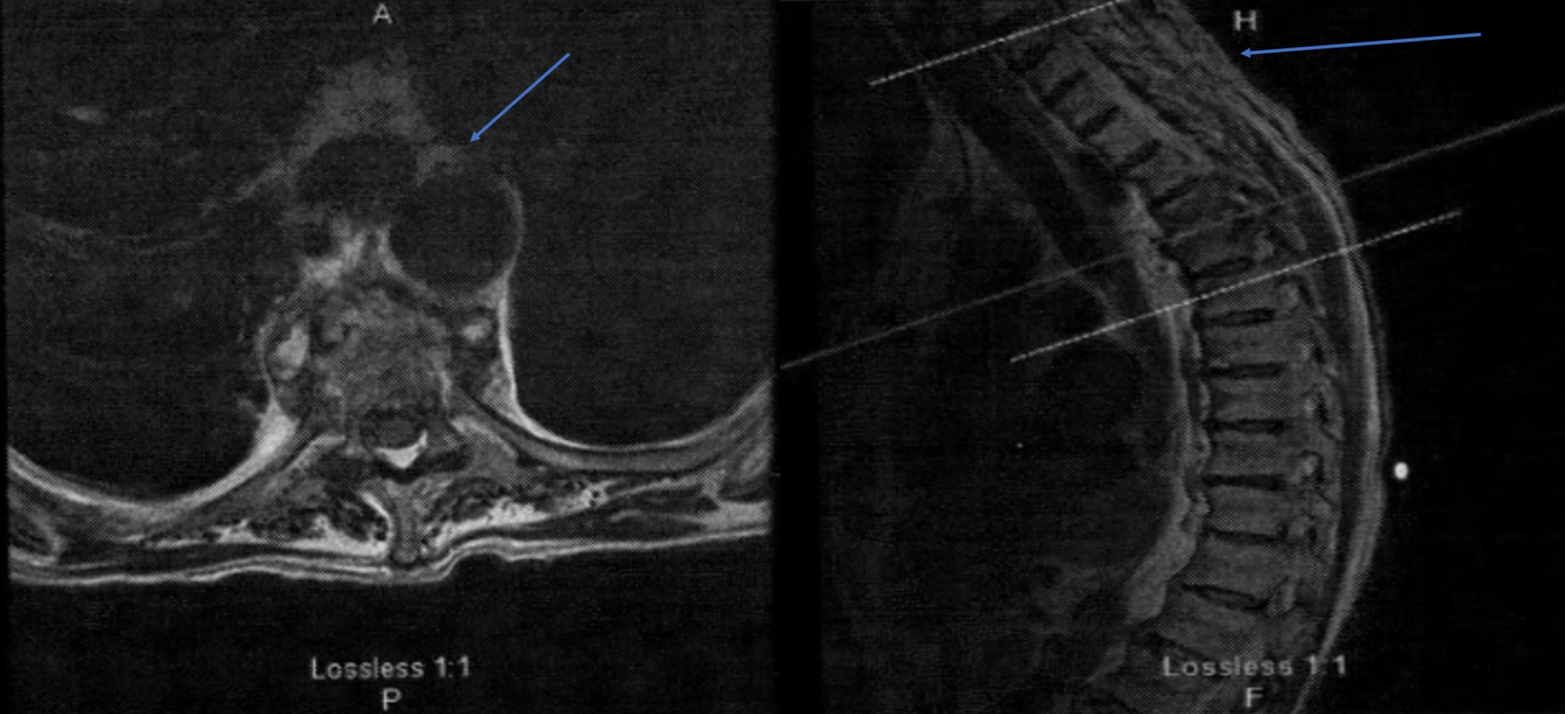
MRI thoracic spine MRI of the thoracic spine showing discitis/osteomyelitis at T4-5 (left) resulting in some elevation of the posterior longitudinal ligament and mild central canal stenosis (right). There was surrounding soft tissue inflammation present anterior and lateral to the spine (left). There was no epidural abscess demonstrated at the time of the exam. The blue arrows demonstrate the findings discussed above

The patient required another hospitalization one week later for recurrent and progressive neck pain with generalized weakness, anorexia, cachexia, and debility without febrility, hypotension, or leukocytosis. MRI of the cervical and thoracic spine with and without contrast revealed discitis and osteomyelitis at T4-T5 with elevation of the posterior longitudinal ligament and mild central canal stenosis with surrounding soft tissue inflammation lateral and anterior to the spine without epidural abscess formation (Figure 2). The patient received treatment with intravenous fortaz and nafcillin with interventional radiology transcutaneous T4-5 disk aspiration under fluoroscopic guidance for bacterial culture. The results of that culture revealed MSSA with identical sensitivities to the two blood cultures, urine cultures, cerebrospinal fluid cultures obtained 22 weeks earlier, two blood cultures obtained 12 weeks earlier, and two blood cultures obtained two weeks earlier. The patient was discharged to a long-term care facility for subacute care to receive six weeks of intravenous cefazolin therapy.

The patient required another hospitalization 13 months later for revision of a right total knee arthroplasty with the insertion of an antibiotic spacer for an infected right total knee prosthesis subsequent to the acute onset of bilateral knee pain with erythema, calor, dolor, and swelling two weeks previously. The right knee synovial fluid culture and intraoperative tissue culture revealed methicillin-resistant *Staphylococcus aureus* (MRSA). Three days later, the patient received a left total knee resection with revision of both arthroplasty prosthesis components and antibiotic articulating spacer insertion. The left knee intraoperative tissue culture revealed MRSA as well. The patient was discharged to a long-term care facility for subacute care to receive six weeks of intravenous vancomycin therapy.

Subsequent to the successful modification of the aberrant behavior of rhinotillexomania and the cessation of the use of over-the-counter intranasal spray products, the patient has not required any further antibiotic therapy or inpatient hospital care.

## Discussion

Over the course of several months, the 66-year-old white female detailed in this case report experienced a potentially life-threatening sequelae of sepsis with acute metabolic encephalopathy and delirium, respiratory failure requiring non-invasive ventilatory support, cystitis, meningitis, endocarditis, pneumonia, discitis, cellulitis, bursitis, and septic arthritis secondary to MSSA. After successful treatment during the two hospital admissions in which blood, urine and CSF cultures were positive for MSSA, the patient was referred to otolaryngology for treatment of her nasal lesions which were found to carry MSSA with identical sensitivities as those from the prior blood, urine and CSF cultures. After adequate counseling and treatment of the nasal lesions, the patient remained culture-negative throughout the remainder of her hospital admissions. The identical culture and sensitivities suggest a common etiology for her widespread systemic infection. In addition, MSSA, being an unusual cause of infection in the organs affected in this patient, suggests spread from a distant site and possibly a more rare and ill-defined etiology for infection. 

Chronic nose-picking has been widely associated with increased bacterial colonization of the anterior nares. The adult nasal microbiota differs between individuals, but species belonging to *Corynebacterium*, *Propionibacterium*, and *Staphylococcus* genera are the most abundant bacteria [7,8]. Although the interactions between various strains of bacteria within the nasal microbiota are complex, a single species generally predominates [7]. In our case, MSSA was the predominant organism that was cultured from the nare of the patient. In 2004, Wertheim et al. showed that *S. aureus* nasal carriers were three times more likely to develop nosocomial *S. aureus* bacteremia compared to non-carriers [9]. Furthermore, 80% of those bacteremic patients demonstrated clonalities identical to their nasal bacterial isolates. Interestingly, Wertheim et al. also demonstrated that although colonization of *S. aureus *was associated with three times greater risk of bacteremia, it did not accompany a greater risk of mortality. In fact, *S. aureus* bacteremia-related death was significantly higher in non-carriers than in carriers [9]. Similarly, the patient detailed in this case developed an infection with a strain of *S. aureus* with identical culture and sensitivity results as those obtained from the nares and did not suffer from bacteremia associated death. It is theorized that exposure of the nasal septal vasculature to the nasal microbiota provided a route for systemic hematogenous spread. 

The complications associated with chronic rhinotillexomania can range from minor to severe and, potentially, life-threatening. Treatment relies on the prevention of extension and progression to greater complications which are often limited by patient access to treatment, patient compliance, and physician accuracy of diagnosis. The condition may initially present as a nosebleed or localized intranasal lesion, but a thorough history and physical examination are essential for early diagnosis and treatment. Once a history of chronic nose-picking has been identified as a potential cause of the patients symptoms, early management should involve counseling the patient on the importance of cessation. While most cases of nose-picking represent a benign habit, when the practice is extreme, as in our patient, it can be a manifestation of an underlying condition. Potential causes may include anxiety, dementia, obsessive-compulsive disorder, empty nose syndrome, allergic or non-allergic rhinitis, and/or anatomical defects [1,10,11]. The goal is to identify and address the underlying etiology whenever possible.

## Conclusions

Although the vast majority of nose-picking is unharmful, the progression to chronic nose-picking has gained some attention in the literature and has been termed rhinotillexomania. After an unusual recurrence of widespread *S. aureus* infection affecting multiple organs as detailed in this case, a thorough history and physical examination revealed a longstanding history of rhinological complaints and nose-picking evidenced by extensive nasal excoriations requiring prompt medical attention. After evaluation and management with otolaryngology, the patient remained culture-negative and was able to experience a full recovery. Thus, we concluded that the patient’s history of nose-picking was a largely preventable risk factor and cause for her illness presentation. Since the identification and diagnosis, in this case, progressed to the point of multidisciplinary involvement, there is a need for otolaryngologists and primary care specialists to educate and counsel patients on the importance of nose-picking cessation in an effort to avoid another case of near-fatal nose-picking.
